# Regulatory RNA: from molecular insights to therapeutic frontiers

**DOI:** 10.1038/s12276-024-01267-2

**Published:** 2024-06-14

**Authors:** TaeSoo Kim, Tae-Kyung Kim

**Affiliations:** 1 Department of Life Science, Ewha Womans University, Seoul, 03760 Republic of Korea; 2 The Research Center for Cellular Homeostasis, Ewha Womans University, Seoul, 03760 Republic of Korea; 3 Multitasking Macrophage Research Center, Ewha Womans University, Seoul, 03760 Republic of Korea; 4 Department of Life Sciences, Pohang University of Science and Technology (POSTECH), Pohang, Gyeongbuk 37673 Republic of Korea; 5 Institute for Convergence Research and Education in Advanced Technology, Yonsei University, Seoul, 03722 Republic of Korea

**Keywords:** Ribosome, RNA decay

Over the years, advancements in modern molecular biology have shed light on the complex roles of RNA molecules. It is now well established that RNAs serve as more than just passive messengers in genetic information flow; rather, they play critical roles in coordinating diverse cellular functions by fine-tuning gene regulation, especially in complex organisms where these processes are intricate^1,2^. Our understanding of gene regulation has undergone a profound transformation with the discovery and characterization of regulatory RNAs.

The genome undergoes pervasive transcription, giving rise to diverse arrays of regulatory RNAs^3^. These can be classified into two classes based on their lengths. Short regulatory RNAs, including microRNAs and small interfering RNAs, are generally less than 200 nucleotides in length. They primarily function through base pairing with complementary sequences in target messenger RNAs (mRNAs), resulting in mRNA degradation or translational repression^4^. Long regulatory RNAs, also called long non-coding RNAs (lncRNAs), are typically longer than 200 nucleotides and range from a few hundred to thousands of nucleotides^5^. They exhibit a wide range of functions and mechanisms of action. Long regulatory RNAs can modulate gene expression at multiple levels, including chromatin modification, transcriptional regulation, and post-transcriptional processing^1^. They serve as scaffolds for assembling protein complexes involved in gene regulation, act as guides for recruiting chromatin-modifying enzymes to specific genomic loci, or function as decoys for binding transcription factors or other regulatory proteins, thereby influencing gene expression^6^. Recently, the significance of circular RNAs (circRNAs) in gene regulation has been increasingly recognized. CircRNAs, which form closed loops, are emerging as important players in gene expression because they can also serve as templates for translation^7,8^. These types of regulatory RNAs are now recognized as pivotal entities that influence various cellular processes. This special issue comprises a series of review articles exploring various facets of regulatory RNAs.

This issue begins with an article on the molecular features of regulatory RNAs as integral cellular machinery. Yang et al.^9^ described a broad functional spectrum of regulatory RNAs that orchestrates transcriptional, post-transcriptional, and epigenetic regulation and underlying molecular mechanisms. This review also highlights the structural alterations that facilitate interactions between various RNA types and other cellular components.

Two articles introduce specific examples of regulatory RNA action in cellular function and development. Alu elements are highly abundant, primate-specific short interspersed nuclear elements whose function has been linked to various stages of gene expression. Lee et al.^10^ introduced a special feature of Alu elements, known as inverted Alu repeats (IRAlus), that can form distinct double-stranded RNAs (dsRNAs) and described their emerging functions, regulatory mechanisms, and implications in immune-related disorders. Yap and Chen^11^ focused on the fascinating topic of miRNA clusters, with a particular focus on the mir-23-27-24 cluster. By elucidating its roles in development and aging, the authors advocate for further exploration of miRNA clusters as key regulators of biological processes throughout the lifespan.

Circular RNAs (circRNAs) are RNA molecules with a covalently closed circular structure. This unique class of RNAs is now known to be present in a wide range of organisms and is increasingly appreciated for its key role in the regulation of gene expression and disease pathology. Generated through a process called back-splicing, circRNAs were initially considered as non-coding RNAs, but recent studies have provided ample evidence that circRNAs can be translated through 5′ cap-independent internal translation initiation. This feature expands the therapeutic applications of circRNAs, and Hwang and Kim^12^ described the key molecular elements that facilitate the translation of circRNAs. They also explored the relationship between circRNA translatability and stability, particularly through mRNA surveillance mechanisms such as nonsense-mediated mRNA decay and nonstop decay. Due to the lack of free ends, circRNAs are generally more stable than linear RNAs. In addition to their known association with disease pathology, this feature makes circRNAs attractive candidates for diagnostic and prognostic biomarkers. Therefore, another exciting prospect of circRNAs in terms of clinical applications is that the synthetic circularization of RNAs could emerge as an application platform for mRNA-based therapies and vaccines. In this regard, Choi and Nam^13^ provided practical guidelines for the optimal design of synthetic circular RNAs and discussed their potential for therapeutic applications.

Finally, Hwang et al.^14^ described the potential of utilizing big data and deep learning to advance research in RNA biology. Their review provides guiding principles for integrating deep learning techniques into RNA biology research, accompanied by discussions on the challenges and strategies associated with the development of deep learning models. This review serves as a valuable introductory resource for researchers in the RNA biology field seeking to incorporate deep learning approaches into their studies.

The discovery of regulatory RNAs not only enriches our understanding of fundamental biological mechanisms but also paves the way for innovative therapeutic intervention and disease treatment. Utilizing the regulatory potential of these RNAs presents exciting opportunities for developing novel therapies aimed at a broad spectrum of diseases. By providing distinctive insights into the diverse facets of regulatory RNAs, these informative reviews will help readers stay abreast of the quickly evolving field of RNA and its transformative impact on modern biology and medicine.
